# How Does Booster Work? A Mediation Analysis of the Effects of Booster Sessions in a Transdiagnostic, Selective, Personalised, Preventive Intervention for At-Risk Youth

**DOI:** 10.1007/s10802-025-01381-3

**Published:** 2025-10-30

**Authors:** David Jimenez-Vazquez, Luis-Joaquin Garcia-Lopez, Laura Zafra-Palomino

**Affiliations:** Department of Psychology, Division of Clinical Psychology, University of Jaen, Jaen, Spain

**Keywords:** Adolescence, Booster sessions, Emotional regulation, Mediation, Prevention, Transdiagnostic

## Abstract

Evidence supports the efficacy of transdiagnostic interventions to prevent emotional problems in adolescents. Recently, the role of booster sessions on treatment gains over time has been suggested. However, the underlying mechanisms that explain treatment outcomes after booster dosage are unknown. This study examined possible mediation effects of booster in a sample of 100 adolescents at risk of developing emotional problems who received the selective, personalised, preventive, transdiagnostic intervention named as PROCARE+. Participants were allocated into three conditions according to the number of booster sessions received after PROCARE + was implemented: none, one (at 6 months) or two (one at 6 and another at 12 months). Mediation models were conducted to examine the role of changes in emotional regulation and resilience on several outcome measures: self-perceived and parent-rated emotional risk, emotional symptomatology and quality of life. Results showed that emotional regulation, but not resilience, significantly mediated the improvements observed after receiving the booster sessions, emerging as a key psychological mechanism. In particular, participants who received one booster session compared to those who received no booster session evidenced improvements in their levels of emotional regulation, which partially mediated the effects on emotional symptomatology and quality of life. In addition to benefits after one booster dosage, adolescents who received two booster dosage also expressed a decrease of their risk of developing emotional problems.

**Supplementary Information:**

The online version contains supplementary material available at 10.1007/s10802-025-01381-3.

## Introduction

Emotional disorders, such as anxiety and depression, are highly prevalent among adolescents (WHO, 2024). These disorders have an early onset (Solmi et al., 2022) and negatively impact multiple areas of adolescents’ lives, including their academic, social, and family functioning (Morales-Muñoz et al., 2023; Wickersham et al., 2021). They may also compromise the transition to adulthood (Alaie et al., 2021; Copeland et al., 2020). In light of this, there is a growing need for research and the implementation of preventive strategies during adolescence (Garcia-Lopez, 2023; Jimenez-Vazquez et al., 2024a; Vivas-Fernandez et al., 2024a). Evidence supports that transdiagnostic interventions in children and adolescents are an effective strategy for addressing emotional problems in prevention settings (Garcia-Lopez et al., 2024; Orgiles et al., 2024; Schmitt et al., 2022; Vivas-Fernandez et al., 2023a, b; Wang et al., 2024).

PROCARE + is a transdiagnostic, selective, personalised preventive intervention specifically designed for adolescents at risk of developing emotional problems (Vivas-Fernandez et al., 2023a). Specifically, PROCARE + is an 8-session, 60-minute, synchronous group program conducted in real time via videoconferencing using Google Meet, allowing for live interaction between participants and therapists. These eight sessions (the core modules) are based on the Unified Protocol for Transdiagnostic Treatment of Emotional Disorders in Adolescents (UP-A; Ehrenreich-May et al., 2018). In addition to the core eight sessions, the personalised intervention incorporates add-on 60-minute modules targeting specific risk factors affecting emotional health during adolescence. These modules are tailored based on the risk factors evidenced by adolescent, such as stress management (including climate and test anxiety), coping with social exclusion and (cyber)victimization, risk of addictive behaviors (including non-substance addiction), and the promotion of healthy habits (including nutrition, sleep, and physical exercise). Further, an add-on family module focused on effective family communication skills is available for families with elevated parental levels of expressed emotion. PROCARE + has demonstrated significantly greater effectiveness than an active control condition at short and long-term, with notable improvements reported in self-perceived and parent-rated risk of emotional problems, anxiety and depression symptoms, emotional regulation, and resilience (Vivas-Fernandez et al., 2023a, b).

Moreover, booster sessions have been increasingly proposed as a means to maintain the long-term effects of cognitive-behavioral therapy (CBT) (Bathgate et al., 2022; Fava et al., 2004; Garcia-Lopez et al., 2024; Gearing et al., 2013; Giovannetti et al., 2021; Johnsen et al., 2024; Mathew et al., 2010; Sun et al., 2019; Zarski et al., 2024). Booster sessions are follow-up sessions delivered after the completion of the main intervention, aimed at reinforcing previously learned skills, preventing relapse, and maintaining long-term treatment effects. These sessions review core content and provide opportunities for skill rehearsal and the application of strategies to new challenges or situations that may arise over time. Building on this, a recent study conducted within a randomized controlled trial (RCT) has examined the differential impact of the number of booster sessions on treatment gains. The study included three experimental groups: an Experimental Group without booster sessions (EG0), a group receiving one booster session at the 6-month follow-up (EG1), and a group receiving two booster sessions at the 6- and 12-month follow-ups (EG2) (Jimenez-Vazquez et al., 2025). Medium- and long-term outcomes were examined following the implementation of the main PROCARE + intervention. Although no significant differences were observed between experimental conditions at the 7-month follow-up based on participation in booster sessions, the findings indicate that attending at least one booster session at the 6-month follow-up is essential to maintain long-term improvements in overall emotional symptoms, emotional regulation, and resilience, compared to receiving no sessions. Furthermore, no significant long-term differences were found between receiving one booster session (EG1) and receiving two booster sessions (EG2). However, participation in a second booster session appeared to provide additional sustained benefits, suggesting that differences between conditions may emerge in longer-term follow-ups.

With regard to previous research examining the mediating mechanisms of the selective transdiagnostic preventive intervention PROCARE, compared to a psychoeducation-based intervention, have shown that resilience mediates both the likelihood of developing emotional problems and quality of life (Jimenez-Vazquez et al., 2024a; Vivas-Fernandez et al., 2024b). However, to date, no research has been conducted to explore the underlying mechanisms that explain the long-term effects of booster sessions following the completion of a transdiagnostic preventive intervention. Studying mediators of treatment could provide information about how or why a given intervention works, and it allows the objectives to be adapted during interventions to increase effectiveness (Kazdin, 2007; Kraemer et al., 2002, 2008).

From a transdiagnostic perspective, the Unified Protocol for the treatment of emotional disorders (Barlow et al., 2017) proposes an intervention focused on common emotional processes present across different forms of emotional psychopathology. Among these processes, emotion regulation is recognized as a fundamental transdiagnostic factor in the development and maintenance of emotional disorders (Sakiris & Berle, 2019). Within this framework, both the core of the preventive intervention PROCARE + and the content of the booster sessions are based on the UP-A (Ehrenreich-May et al., 2018), an adaptation of the Unified Protocol for adolescents. This protocol incorporates techniques and tools aimed at improving emotion regulation skills and reducing dysfunctional patterns such as avoidance, suppression, or emotional intensification (Ehrenreich-May et al., 2015; Trosper et al., 2009). PROCARE + also includes personalised modules designed to target previously identified emotional risk factors in participants, with the goal of strengthening, alongside emotion regulation, resilience, understood as a protective resource against the development of emotional symptoms and as a key facilitator of adaptation to adversity (Jimenez-Vazquez et al., 2024a; Pan et al., 2024; Vivas-Fernandez et al., 2024b). Therefore, emotion regulation and resilience are considered central therapeutic processes in this model. In this regard, resilience is defined as the capacity to adapt to stress and adversity, being a dynamic process through which individuals positively adjust to challenging or traumatic circumstances (Connor & Davidson, 2003; Luthar et al., 2000). It has been suggested that resilience is involved in alleviating emotional distress and reducing certain risky behaviours, as well as promoting more adaptive coping strategies (Cai et al., 2023; Ng et al., 2012; Mętel et al., 2019; Shang et al., 2024; Sher et al., 2019). Specifically, in adolescents, resilience is associated with lower vulnerability to anxious-depressive symptoms or stress (Hjemdal et al., 2011). Likewise, mediating effects of resilience on well-being and psychological distress have been identified following psychological interventions in adolescent populations (Jimenez-Vazquez et al., 2024b; Schweickle et al., 2024).

Similarly, emotion regulation, the processes responsible for monitoring, evaluating, and modifying emotional reactions to achieve goals (Gross, 1998; Koole, 2009; Thompson, 1994), has been established as a key mechanism in the onset of anxious-depressive symptoms (Lin et al., 2024; Lincoln et al., 2022), and it is strongly related to emotional well-being and perceived self-efficacy in young people, as well as improvements in certain healthy lifestyle habits, such as sleep (Üstündağ, 2024). In other cases, it has been linked to problems such as childhood post-traumatic stress disorder and adult depression, where emotional regulation also plays a key role (Hopfinger et al., 2016). A substantial body of empirical research has shown that interventions directly targeting emotion regulation exert a significant mediating effect on the reduction of anxiety and depressive symptoms, thereby underscoring its clinical importance (Abasi et al., 2021; Barrio-Martínez et al., 2022; Khakpoor et al., 2019; Slee et al., 2008).

Until now, the mediating effects associated with the implementation of booster sessions have not been explored in terms of their benefits and advantages, either in the context of traditional cognitive-behavioral therapy (CBT) interventions or in transdiagnostic approaches targeting adolescents. In light of previous evidence identifying resilience and emotional regulation as key variables in understanding and addressing emotional problems in adolescence, this study aims to analyse the mediation mechanisms associated with the observed benefits of incorporating booster sessions into the personalised PROCARE + selective, personalised, preventive intervention. In particular, we will assess whether emotional regulation and/or resilience may act as mediating variables in the positive long-term effects observed when participants: (1) participating in a booster session at the 6-month follow-up compared to not participating in a booster session; (2) receiving two booster sessions at the 12- and 6-month follow-ups compared to not receiving a booster session; or (3) receiving two booster sessions at the 12- and 6-month follow-ups compared to receiving a single booster session at the 6-month follow-up. To this end, we will examine the possible mediating impact on the following outcome variables: self-perceived and parent-rated level of risk of emotional difficulties, anxiety and depressive symptomatology scores, and quality of life.

## Method

### Participants

The sample consisted of 100 adolescents (52% self-identified females), aged between 12 and 18 years (*M* = 13.46; *SD* = 1.28), who participated in an RCT (Jimenez-Vazquez et al., 2025). Participants met the following inclusion criteria: (1) written informed consent from the adolescent and their legal guardian; (2) linguistic competence, defined as the adequate ability to comprehend and communicate effectively in the language used during the intervention and assessments, enabling independent completion of tasks and participation in sessions; (3) having the necessary technological resources, including a device capable of videoconferencing to complete the assessments and attend online sessions; (4) being between 12 and 18 years old; (5) not receiving concurrent psychological/psychiatric treatment; (6) not having a diagnosis of autism spectrum disorder or attention-deficit/hyperactivity disorder; and (7) being at risk of future emotional disorders, based on gender-specific criteria and the algorithm by Piqueras et al. (2024), which specifies that the selective prevention group must exhibit moderate resilience scores on the CD-RISC-10 (Lopez-Fernandez et al., 2024), low to moderate risk factors and emotional symptoms on the Spanish version of the SDQ emotional symptoms subscale (self- or parent-reported) (Ortuño-Sierra et al., 2022; Rodriguez-Hernandez et al., 2014), and low overall emotional symptomatology or scores below normative thresholds on any subscale of the RCADS-30 (Pineda et al., 2018). All eligible participants who met the inclusion criteria benefitted from the PROCARE + active treatment intervention. After the intervention, the participants were randomly assigned to one of three experimental conditions to study the effects of booster sessions: (i) Experimental Group 0, without booster sessions (EG0; *n* = 21); (ii) Experimental Group 1, with one booster session at the 6-month follow-up (EG1; *n* = 40); (iii) Experimental Group 2, with two booster sessions —one at 6 months and another at 12 months (EG2; *n* = 39). An intention-to-treat (ITT) analysis revealed that there were no statistically significant differences (*p* >.05) between those eligible for treatment and randomized to booster assignment but refused to participate in the trial compared to those who completed the booster sessions. Further, no statistically significant differences on age, gender, nationality, or session attendance between experimental conditions were found. Further details can be found in Jimenez-Vazquez et al. (2025). Table 1

**Table 1:** Sociodemographic data

		G0: No booster *n* = 21	G1: 1 booster *n* = 40	G2: 2 booster *n* = 39	Statistical test
Age	*M* (*SD*)	13.62 (1.43)	13.45 (1.34)	13.38 (1.14)	*H* (2) = 0.29; *p* =.86
	*n* (%)				
Gender					
Female		14 (66.67%)	20 (50%)	18 (46.15%)	χ² (2) = 2.41; *p* =.30
Male		7 (33.33%)	20 (50%)	21 (53.85%)	
Nationality					
Spanish		20 (95%)	35 (87.5%)	36 (92.3%	*LR* (2) = 1.17; *p* =.56
Not Spanish		1 (0.5%)	5 (12.5%)	3 (7.7%)	

*M * Mean; *SD* Standard Deviation; *ns*: non-significant *p* >.05

### Measures

All assessments were conducted at various time points, from pre-treatment to the 12-month post-intervention follow-up. These were carried out using the secure Google Forms platform. The relevant outcome measures for the mediation analyses were as follows:

- The Strengths and Difficulties Questionnaire
- (SDQ; Goodman, 1997), available at www.sdqinfo.org, is composed of 25 items rated on a 3-point Likert scale (0 = not true, 1 = somewhat true, 2 = certainly true). It evaluates emotional and behavioral problems, hyperactivity/inattention, peer relationship problems, and prosocial behavior in children and adolescents. The instrument has been adapted into multiple languages, Spanish among them, and shows strong cross-cultural validity. Both its self-report and parent-report formats have shown solid psychometric properties and established cut-off scores suitable for screening (Ortuño-Sierra et al., 2022; Rodríguez-Hernández et al., 2014). For the current study, the 5-item emotional symptoms subscale from both the parent-report version (SDQ-P) and the self-report version (SDQ-A) was utilized to assess emotional risk (Armitage et al., 2023). This study found good reliability for both self-report (α = 0.81, ω = 0.82) and parent versions (α = 0.80, ω = 0.81).

- The 30-item version of the Revised Child Anxiety and Depression Scale
- (RCADS-30; Sandín et al., 2010) includes 30 statements rated on a 4-point Likert scale ranging from 0 (never) to 3 (always). This instrument is designed to evaluate symptoms of anxiety and depression in children and adolescents through different subscales (panic disorder, social phobia, separation anxiety disorder, generalized anxiety disorder, obsessive-compulsive disorder and major depressive disorder. It has excellent psychometric properties for Spanish populations (Pineda et al., 2018). In this study, the total RCADS-30 score was employed as a global indicator of anxiety and mood-related symptoms. The scale demonstrated excellent internal consistency (Cronbach’s alpha, α = 0.94; McDonald’s omega, ω = 0.94).

- The KIDSCREEN-10 Index
- (Ravens-Sieberer et al., 2001) is a self-report measure designed to evaluate health-related quality of life in children and adolescents, covering dimensions related to physical, emotional, and social well-being. It includes 10 items, with a Likert-type response format ranging from 0 to 5 (not at all, a little, moderately, a lot and very much). The psychometric properties are adequate (Ravens-Sieberer et al., 2010). In this study, the Cronbach’s alpha value was 0.84 and the McDonald’s omega was 0.84, consistent with previous evidence from Spanish-speaking adolescent populations (Vivas-Fernandez et al., 2023a, b; Garcia-Lopez et al., 2024).

The measures used as mediator variables in the analyses included:

- The Difficulties in Emotion Regulation Scale
- (DERS; Gratz & Roemer, 2004) was employed in its Spanish adaptation (Hervás & Jódar, 2008). This instrument assesses difficulties in emotion regulation through 36 items, each rated on a 5-point Likert scale ranging from 0 (almost never) to 4 (almost always). The items are distributed across six core dimensions: (1) non-acceptance of emotional responses, (2) difficulty engaging in goal-directed behavior when distressed, (3) problems controlling impulsive behaviors under emotional pressure, (4) limited access to effective emotion regulation strategies, (5) lack of emotional awareness, and (6) poor emotional clarity. In the present study, the scale demonstrated excellent internal consistency, with a Cronbach’s alpha of 0.94 and a McDonald’s omega of 0.94.

- The 10-Item Connor-Davidson Resilience Scale
- (CD-RISC-10; Campbell-Sills & Stein, 2007). It is the self-report shortened version of the original the Connor-Davidson Resilience Scale (Connor & Davidson, 2003) which assesses resilience through 10 items with a Likert-type response scale ranging from 0 to 4 (not at all, rarely, sometimes, often, and almost always. The Spanish version, which has shown strong psychometric properties, is widely regarded as a reliable and valid tool for assessing resilience (Lopez-Fernandez et al., 2024). In this study, reliability was also good (α = 0.87, ω = 0.87).

### Procedure

The sample was recruited mainly through secondary schools in Spain. In addition, in order to ensure the representativeness of the sample was broad, the study was also disseminated through social networks and supported by government institutions, non-governmental organisations (NGOs) and third sector organisations. Informed consent was obtained in March 2023 from both legal guardians and the adolescents themselves (or from the adolescents only if they were aged 16 or over, in accordance with Spanish legislation).

The pre-test assessment allowed for the identification of adolescents evidencing risk of emotional difficulties, as well as served as a baseline for evaluating the changes experienced by participants throughout the PROCARE + intervention.

**Fig. 1:**
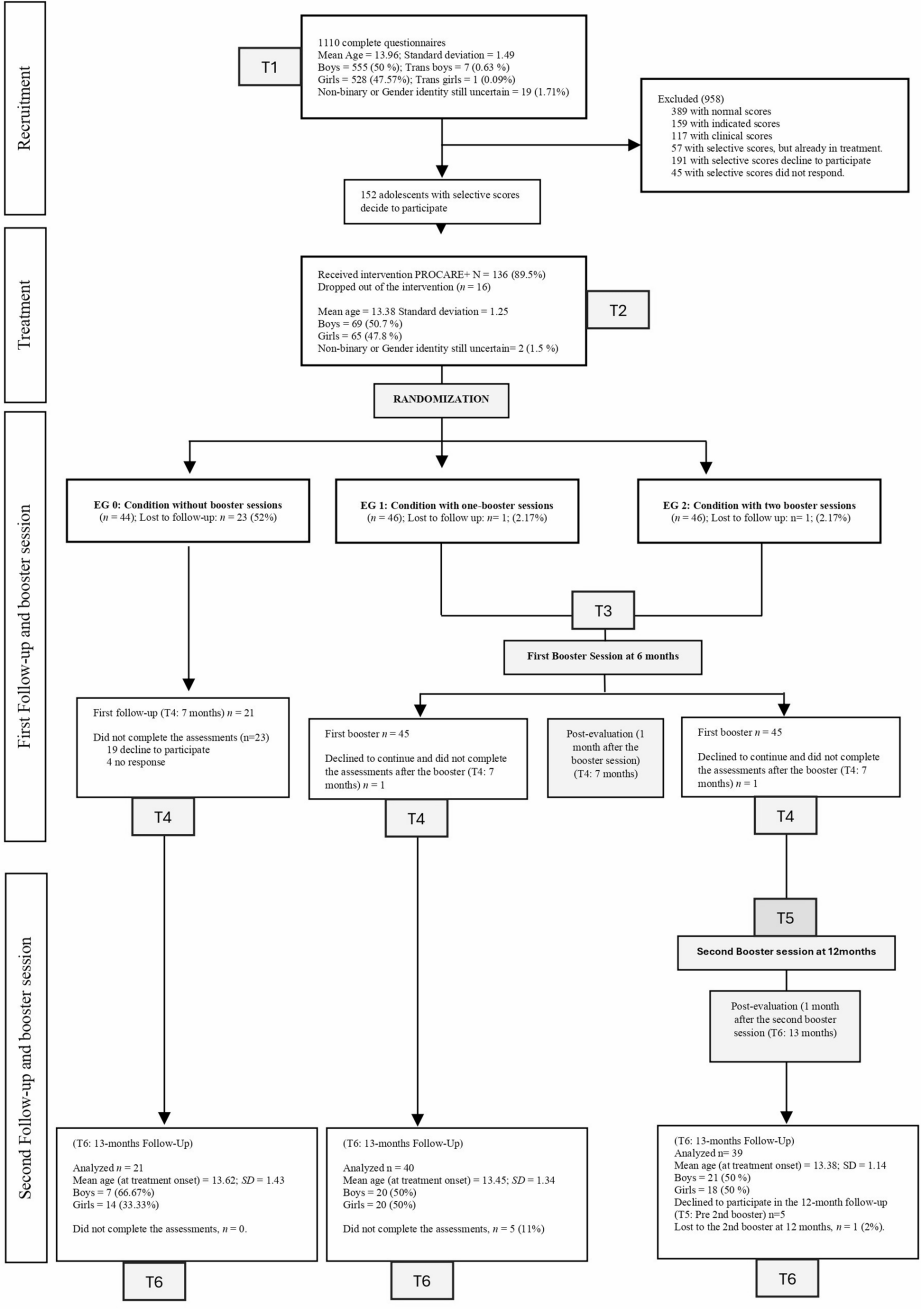
Consort flow diagram

Participants in the study received the main PROCARE + intervention (including both general modules and add-on modules, tailored on the presence of risk factors). After completing the main intervention, participants were randomly assigned to one of three experimental conditions for the differential study of the long-term effects of booster sessions, which consisted of a 90-minute review of the PROCARE + content. These sessions provide the opportunity to practice skills and strategies in new challenges and to prevent relapse. No differences were found among participants in the three experimental conditions at either the pre-test or the post-test following the main intervention. For EG0, a follow-up assessment was conducted 13 months after the main intervention. For EG1, a booster session was delivered 6 months after the main intervention, followed by a follow-up assessment at 13 months. Finally, EG2 received two booster sessions—at 6 and 12 months—and a follow-up assessment at 13 months after the main intervention (see Fig. 1).

The study followed the Consolidated Standards of Reporting Trials (CONSORT: http://www.consort-statement.org) and the SPIRIT guidelines (Standard Protocol Items: Recommendations for Interventional Trials). Additionally, it was registered in the ClinicalTrials.gov database under the identifier NCT06185049. Institutional review board (IRB) approval was obtained, and all evaluations were conducted online via a secure platform. This research was authorized by the bioethics committee of the University of Jaen (ID: GEN-3461-aab8-41a3-85c2-ca28-5102-cdda-8d53). The trial was planned according to internationally adopted guidelines (ICH-E6, E8, and E9), and in accordance with other guidelines, such as those from the European Medicines Agency (EMA), as well as complying with current data protection legislation (Regulation (EU) 2016/679).

### Data Analysis

First, we checked that there were no differences in the sociodemographic and clinical characteristics of the sample, and that the experimental groups were homogeneous at pre-treatment and post-treatment. To verify the regression assumptions, complementary analyses were conducted in SPSS. Normality and homoscedasticity of the residuals were assessed using residual plots and Q-Q plots, while multicollinearity was examined through VIF values, which remained below 10 in all models. Second, following the recommendations of Preacher and Hayes (2004), mediation models were used to determine the extent to which a change in emotional regulation and resilience after receiving or not receiving booster sessions explained the change in the outcome variables (self-reported emotional risk, as measured by the Emotional Risk Subscale, measured by the Emotional Subscale of the SDQ-A, parent-reported emotional risk assessed by the Emotional Subscale of the SDQ-P, general anxiety and mood symptomatology assessed by the RCADS-30, and quality of life measured by the KIDSCREEN-10).

In this sense, four mediation models were established to assess whether changes in each outcome variable are mediated by improvements in emotional regulation and/or resilience when comparing EG1 with EG0 (i.e. one versus no booster session); four to compare EG2 with EG0 (two versus no sessions); and four to compare EG2 with EG1 (two versus one session). The mediator and dependent variables in these models were the residualized change scores, calculated through linear regression between the pre-intervention assessment (T1) and the evaluation conducted 13 months after the main intervention (T6) across the three experimental conditions (Collins & Horn, 1991). This procedure has been previously used as a reliable method to control for the variability of baseline scores (Nieto & Vázquez, 2021; Sanchez et al., 2016; Segal et al., 2006).

We used the SPSS PROCESS macro (version 4.3) to test the mediation effects (Hayes, 2017). In these analyses, unstandardized coefficients, standard errors, and lower and upper limits were examined. The macro provides bias-corrected 95% confidence intervals for the indices using nonparametric bootstrap calculations, based on 10,000 samples. Significance is reached when the estimated 95% bootstrap confidence intervals do not contain 0. Data analyses were conducted using SPSS (version 28.0).

## Results

### Mediation Models: One Booster Session Vs. No Booster Session

Table 2 presents the results of four mediation models (Models 1–4) in which we analyzed whether changes in emotional regulation and resilience mediated the relationship between Experimental Group (experimental condition with 1 booster session, at 6 follow-up, versus the condition with no booster session after the main intervention, i.e., EG1 vs. EG0) and the outcomes variables: change in self-reported emotional risk, parent-reported emotional risk, anxiety and depression symptoms and quality of life.

**Table 2:** Mediation models: changes in emotional risk (Self- and Parent-Reported), emotional Symptomatology, and quality of life (Without booster session vs. One booster sessions)

Model	X	M_1_	M_2_	Y	Total Effect c			Direct Effect c’			a			b		Indirect Effect a x b	
					*R* ^*2*^	*ᵝ (SE)*	*t (p)*	*ᵝ (SE)*	*t (p)*		*ᵝ* (SE)	*t (p)*		*ᵝ* (*SE*)	*t (p)*	*ᵝ (SE)*	95% CI
1	EG	DERS	CD-RISC-10	SDQ-A	0.24	−1.15 (0.26)	− 4.38 (0.001***)	−0.90 (0.26)	− 3.43 (0.001**)	a_1_	−0.67 (0.25)	−2.66 (0.01**)	b_1_	0.40 (0.12)	3.11 (0.003**)	−0.27 (0.19)	[−0.07, 0.01]
										a_2_	0.29 (0.25)	1.16 (0.24)	b_2_	−0.69 (0.13)	0.54 (0.58)	−0.02 (0.06)	[−0.08, 0.16]
2	EG	DERS	CD-RISC-10	SDQ-P	0.19	−1.04 (0.28)	−3.75 (0.001***)	−1.01 (0.3)	−3.36 (0.001**)	a_1_	−0.67 (0.25)	−2.66 (0.01**)	b_1_	0.50 (0.14)	0.33 (0.73)	−0.03 (0.01)	[−0.23, 0.15]
										a_2_	0.29 (0.25)	1.16 (0.24)	b_2_	−0.03 (0.14)	−0.02 (0.98)	−0.01 (0.04)	[−0.08, 0.11]
3	EG	DERS	CD-RISC-10	RCADS	0.17	−0.87 (0.24)	−3.59 (0.001***)	−0.46 (0.20)	− 2.21 (0.03*)	a_1_	−0.67 (0.25)	−2.66 (0.01**)	b_1_	0.55 (0.10)	5.44 (0.001***)	−0.37 (0.16)	[−0.73, −0.09]
										a_2_	0.29 (0.25)	1.16 (0.24)	b_2_	0.12 (0.10)	−1.15 (0.25)	−0.03 (0.05)	[−0.16, 0.05]
4	EG	DERS	CD-RISC-10	KIDSCREEN	0.08	0.62 (0.27)	2.22 (0.003*)	0.28 (0.27)	1.04 (0.30)	a_1_	−0.67 (0.25)	−2.66 (0.01**)	b_1_	−0.41 (0.13)	−3.05 (0.003**)	0.27 (0.16)	[0.02, 0.67]
										a_2_	0.29 (0.25)	1.16 (0.24)	b_2_	−0.22 (0.13)	−1.64 (0.11)	−0.06 (0.12)	[−0.07, 0.38]

X = Independent variable: Experimental Group (EG: without booster session vs. two booster session), M_1_ = mediator variable: Difficulties in Emotion Regulation Scale (DERS), M_2_ = mediator variable: The 10-Item Connor-Davidson Resilience Scale (CD-RISC-10); Y = outcome variable: Emotional subscale Self-Report SDQ, The Strengths and Difficulties Questionnaire Adolescents (SDQ-A), Emotional subscale Parent SDQ, The Strengths and Difficulties Questionnaire Parents (SDQ-P), The Revised Child Anxiety and Depression Scale (RCADS), KIDSCREEN-10 Index (KIDSCREEN)

*a*: path a; *b*: path b; *ᵝ*: Unstandardized coefficients; *SE*: Standard Error; CI: Confidence interval; *p* <.05*, *p* <.01**, *p* <.001***

10.000 bootstrap samples

For these comparisons between EG1 and EG0 only model 3 and 4 showed mediation effects. Specifically, for model 3, the results showed only a statistically significant indirect effect (*a1* x *b1*) when analysing the mediation of the variable change in DERS on the change in RCADS-30. Given that the sign of the *β1* coefficient is negative, participating in the condition receiving a booster session at 6-month follow-up reduced the anxiety and mood symptomatology by RCADS-30 to a greater extent and these effects are mediated by improvement in emotional dysregulation (*β1* = −0.37, *SE* = 0.16, 95% CI [−0.73, −0.09]). In addition, the results showed a partial mediation effect of emotional dysregulation given the significant result of the direct effect of the experimental condition on the change in the anxiety and mood symptomatology by RCADS-30 (*c’* = −0.46, *SE* = 0.20; *p* =.03).

Regarding model 4, the results again showed a statistically significant indirect effect (*a1* x *b1*) when analysing the mediation of change in DERS on change in KIDSCREEN-10. Given the positive sign of the *β1* coefficient, participating in the condition receiving a booster session at 6-month follow-up allowed for greater quality of life by KIDSCREEN-10 and these effects were mediated by improved emotional dysregulation (*β1* = 0.27, *SE* = 0.16, 95% CI [−0.02, 0.67]). Thus, the booster session condition reduced emotional dysregulation (*a1* = −0.67, SE = 0.25); *p* =.01) and these improvements in emotional regulation improved quality of life (*b* = −0.41, *SE* = 0.13; *p* =.003).

The mediating variable changes in resilience did not produce mediation effects in any of the models presented and, furthermore, no mediation effects were obtained in model 1 and 2, i.e. on self-reported and parent-reported emotional risk, although there was a direct effect of the experimental condition.

### Mediation Models: Two Booster Sessions Vs. No Booster Session

Table 3 presents the results of the four mediation models (Modes 5–8) in which we analysed whether changes in emotional regulation and resilience mediated the relationship between Experimental Group (experimental condition with 2 booster sessions, at 6- and 12-months follow-up, versus the condition with no booster session after the main intervention, i.e., EG2 vs. EG0) and the outcomes variables.

**Table 3:** Mediation models: changes in emotional risk (Self- and Parent-Reported), emotional Symptomatology, and quality of life (Without booster session vs. two booster sessions)

Model	X	M_1_	M_2_	Y	Total Effect c			Direct Effect c’			a			b		Indirect Effect a x b	
					*R* ^*2*^	*ᵝ (SE)*	*t (p)*	*ᵝ (SE)*	*t (p)*		*ᵝ* (SE)	*t (p)*		*ᵝ* (*SE*)	*t (p)*	*ᵝ (SE)*	95% CI
5	EG	DERS	CD-RISC-10	SDQ-A	0.35	−1.30 (0.23)	− 5.57 (0.001***)	−0.83 (0.22)	− 3.74 (0.001***)	a_1_	−0.77 (0.27)	2.83 (0.006**)	b_1_	0.47 (0.10)	4.75 (0.001***)	−0.36 (0.19)	[−0.80, −0.08]
										a_2_	1.02 (0.25)	4.04 (0.001***)	b_2_	−0.12 (0.11)	−1.13 (0.26)	−0.12 (0.12)	[−0.38, 0.07]
6	EG	DERS	CD-RISC-10	SDQ-P	0.33	−1.14 (0.21)	−5.30 (0.001***)	−1.08 (0.25)	−4.30 (0.001***)	a_1_	−0.77 (0.27)	2.83 (0.006**)	b_1_	0.40 (0.11)	0.35 (0.72)	−0.03 (0.10)	[−0.23, 0.17]
										a_2_	1.02 (0.25)	4.04 (0.001***)	b_2_	−0.03 (0.12)	−0.23 (0.82)	−0.03 (0.11)	[−0.26, 0.21]
7	EG	DERS	CD-RISC-10	RCADS	0.24	−1.03 (0.24)	− 4.31 (0.001***)	−0.61 (0.22)	− 2.76 (0.008**)	a_1_	−0.77 (0.27)	2.83 (0.006**)	b_1_	0.54 (0.10)	5.55 (0.001***)	−0.42 (0.18)	[−0.81, −0.11]
										a_2_	1.02 (0.25)	4.04 (0.001***)	b_2_	0.01 (0.10)	−0.08 (0.93)	−0.01 (0.10)	[−0.24, 0.18]
8	EG	DERS	CD-RISC-10	KIDSCREEN	0.22	1.03 (0.25)	4.06 (0.001***)	0.88 (0.26)	3.32 (0.001**)	a_1_	−0.77 (0.27)	2.83 (0.006**)	b_1_	−0.46 (0.11)	−3.88 (0.001***)	0.35 (0.18)	[0.07, 0.79]
										a_2_	1.02 (0.25)	4.04 (0.001***)	b_2_	−0.19 (0.12)	−1.52 (0.14)	−0.20 (0.12)	[−0.47, 0.18]

X = Independent variable: Experimental Group (EG: without booster session vs. two booster session), M_1_ = mediator variable: Difficulties in Emotion Regulation Scale (DERS), M_2_ = mediator variable: The 10-Item Connor-Davidson Resilience Scale (CD-RISC-10); Y = outcome variable: Emotional subscale Self-Report SDQ, The Strengths and Difficulties Questionnaire Adolescents (SDQ-A), Emotional subscale Parent SDQ, The Strengths and Difficulties Questionnaire Parents (SDQ-P), The Revised Child Anxiety and Depression Scale (RCADS), KIDSCREEN-10 Index (KIDSCREEN)

*a*: path *a*; *b*: path *b*; *ᵝ*: Unstandardized coefficients; *SE*: Standard Error; CI: Confidence interval; *p* <.05*, *p* <.01**, *p* <.001***

10.000 bootstrap samples

For model 5, results showed only a statistically significant indirect effect (*a1* x *b1*) when analysing the mediation of the change in DERS on the change in the Emotional subscale of self-reported SDQ. Given that the sign of the β1 coefficient is negative, participating in the condition receiving two booster sessions reduced adolescents’ self-reported emotional risk as assessed by the change in the Emotional subscale of self-reported SDQ to a greater extent, and these effects were mediated through a reduction in emotional dysregulation (*β1* = −0.36, *SE* = 0.19, 95% CI [−0.80, −0.08]). In addition, the results showed a partial mediation effect of emotional dysregulation given the significant result of the direct effect of the experimental condition on the change on the Emotional subscale of self-reported SDQ (*c’*= 0.83, *SE* = 0.22; *p* =.001).

Regarding model 7, the results again showed only a statistically significant indirect effect (*a1* x *b1*) when analysing the mediation of the change in emotional dysregulation in DERS on the anxiety and mood symptomatology by RCADS-30. The negative sign of the *β1* coefficient indicated that participating in the condition receiving two booster sessions reduced the anxiety and mood symptomatology to a greater extent and these effects are mediated by a reduction in emotional dysregulation (*β1* = −0.42, *SE* = 0.18, 95% CI [−0.81, −0.11]). The results showed that the mediation effect of the improvement in emotional dysregulation was a partial mediation effect, given the significant result of the direct effect of the experimental condition on the change in the anxiety and mood symptomatology (*c’* = 0.61, *SE* = 0.22; *p* =.008).

Concerning model 8, the results show a statistically significant indirect effect (*a1* x *b1*) when we analysed the mediation of the change in emotional dysregulation in the DERS on the change in quality of life in the KIDSCREEN-10. Participating in the condition receiving two booster sessions achieved higher quality of life and these effects were mediated by improvement in emotional dysregulation (*β1* = 0.35, *SE* = 0.18, 95% CI [−0.07, 0.79]). On this occasion, the results showed that the effect of mediation by the improvement in emotional dysregulation is partial, given the significant result of the direct effect of the experimental condition on the change in quality of life (*c’* = 0.88, *SE* = 0.26; *p* =.001).

Again, the mediating variable changes in resilience did not produce mediation effects in any of the models presented, and, furthermore, for model 6 no mediation effects were observed on parent-reported emotional risk (assessed by the change in Emotional subscale of SDQ-P).

### Mediation Models: Two Booster Sessions Vs. One Booster Session

Table 4 presents the results of the four mediation models (Models 9–12) in which we examined whether there are effects of mediation by changes in emotional dysregulation and resilience on the established outcome measures when comparing the experimental condition with 2 booster sessions, at 6- and 12-months follow-up versus the condition with a single booster session at 6 months follow-up (EG2 versus EG1).

**Table 4:** Mediation models: changes in emotional risk (Self- and Parent-Reported), emotional Symptomatology, and quality of life (EG1: one booster session vs. EG2: two booster sessions)

Model	X	M_1_	M_2_	Y	Total Effect c			Direct Effect c’			a			b		Indirect Effect a x b	
					*R* ^*2*^	*ᵝ (SE)*	*t (p)*	*ᵝ (SE)*	*t (p)*		*ᵝ* (SE)	*t (p)*		*ᵝ* (*SE*)	*t (p)*	*ᵝ (SE)*	95% CI
9	EG	DERS	CD-RISC-10	SDQA-E	0.01	−0.15 (0.17)	−0.90 (0.37)	−0.12 (0.16)	− 0.77 (0.44)	a_1_	−0.10 (0.21)	−0.68 (0.47)	b_1_	0.24 (0.09)	2.51 (0.01*)	−0.023 (0.06)	[−0.15, 0.07]
										a_2_	0.11 (0.21)	0.54 (0.58)	b_2_	−0.04 (0.09)	−0.40 (0.96)	−0.004 (0.02)	[−0.05, 0.04]
10	EG	DERS	CD-RISC-10	SDQP-E	0.001	−0.09 (0.19)	−0.48 (0.62)	−0.08 (0.20)	−0.42 (0.67)	a_1_	−0.10 (0.21)	−0.68 (0.47)	b_1_	0.05 (0.11)	0.51 (0.61)	−0.002 (0.03)	[−0.07, 0.04]
										a_2_	0.11 (0.21)	0.54 (0.58)	b_2_	−0.03 (0.11)	−0.34 (0.73)	−0.005 (0.03)	[−0.05, 0.06]
11	EG	DERS	CD-RISC-10	RCADS	0.007	−0.17 (0.21)	− 0.74 (0.45)	−0.08 (0.17)	− 0.48 (0.63)	a_1_	−0.10 (0.21)	−0.68 (0.47)	b_1_	0.53 (0.10)	5.37 (0.000*)	−0.05 (0.11)	[−0.31, 0.16]
										a_2_	0.11 (0.21)	0.54 (0.58)	b_2_	−0.21 (0.10)	−2.18 (0.03*)	−0.02 (0.05)	[−0.13, 0.07]
12	EG	DERS	CDRISC	KIDSCREEN	0.06	0.41 (0.18)	2.23 (0.028*)	0.40 (0.18)	2.22 (0.029*)	a_1_	−0.10 (0.21)	−0.68 (0.47)	b_1_	−0.24 (0.10)	−2.37 (0.02*)	0.02 (0.06)	[−0.07, 0.17]
										a_2_	0.11 (0.21)	0.54 (0.58)	b_2_	−0.11 (0.10)	−1.09 (0.27)	−0.01 (0.03)	[−0.08, 0.04]

X = Independent variable: Experimental Group (EG: without booster session vs. two booster session), M_1_ = mediator variable: Difficulties in Emotion Regulation Scale (DERS), M_2_ = mediator variable: The 10-Item Connor-Davidson Resilience Scale (CD-RISC-10); Y = outcome variable: Emotional subscale Self-Report SDQ, The Strengths and Difficulties Questionnaire Adolescents (SDQA), Emotional subscale Parent SDQ, The Strengths and Difficulties Questionnaire Parents (SDQP), The Revised Child Anxiety and Depression Scale (RCADS), KIDSCREEN-10 Index (KIDSCREEN)

*a*: path a; *b*: path b; *ᵝ*: Unstandardized coefficients; *SE*: Standard Error; CI: Confidence interval; *p* <.05*, *p* <.01**, *p* <.001***

10.000 bootstrap samples

No mediation effects were observed for either changes in emotion dysregulation or resilience on the outcome variables when comparing the condition with two boosters versus the condition with one booster session. However, model 12 which examined mediation effects on the outcome variable changes in quality of life, revealed a total effect of the experimental condition on change in quality-of-life *β* = 0.41, *SE* = 0.18, *t* = 2.23, *p* =.028. The direct effect *c’* was significant after controlling for the effects of the mediating variables *β* = 0.40, *SE* = 0.18, *t* = 2.22, *p* =.029, indicating that the condition with two booster sessions was associated with higher quality of life compared to the condition receiving only one.

## Discussion

The present study examined the mediating mechanisms underlying the improvements after the implementation of booster sessions, within the framework of a preventive transdiagnostic intervention for adolescents at risk of emotional difficulties named as PROCARE+. In particular, the study examined whether the number of booster sessions produced improvements in emotional regulation and/or resilience that mediated the long-term outcomes of the PROCARE preventive intervention, as assessed by self- and parent-reported levels of risk of developing emotional difficulties, anxiety and depression symptomatology, and quality of life. Specifically, we assessed whether. Specifically, we assessed whether these variables mediate the effects of receiving two booster sessions at 6- and 12-month follow-ups compared to receiving no booster session or a single booster session at the 6-month follow-up, as well as the mediation effects of receiving a single booster session at 6 months versus receiving no booster session. In line with previous research (Garcia-Lopez et al., 2024; Jimenez-Vazquez et al., 2025; Vivas-Fernandez et al., 2023a, b), the results of the present research suggest a possible mediating role for the improvement of emotional regulation, but not for resilience, after the application of the booster sessions within a transdiagnostic preventive intervention. Previous literature has explored the role of both constructs - emotional regulation and resilience - as possible mechanisms of change in psychological interventions targeting different mental health difficulties. In particular, recent studies indicate that changes in resilience may play a key role in improving outcomes following participation in transdiagnostic, personalised preventive interventions, especially when compared to approaches focused solely on psychoeducation (Jimenez-Vazquez et al., 2024a; Vivas-Fernandez et al., 2024b). However, the data from the present study suggest that the benefits resulting from the booster sessions are not mediated by an increase in resilience, but rather by gains in emotional regulation.

Data revealed that participating in a booster session at 6-month follow-up was associated with improved global emotional symptomology scores compared to not receiving any booster session. This effect was partially mediated by improvements in emotional regulation. Similarly, the observed improvements in quality-of-life in this group are completely mediated by emotional regulation. Participation in two booster sessions at 6- and 12-month follow-ups compared to absence of booster sessions was associated with a direct effect on global emotional symptomatology scores, self-perceived emotional risk, and quality of life. These results suggest that adding a second booster session at 12 months could provide additional benefits, reinforcing the importance of ongoing structured support to sustain and enhance the gains made with the initial intervention. However, these effects are also partially mediated by improvements in emotional regulation. Therefore, while participation in the booster sessions appears to directly impact the outcome variables, emotional regulation emerges as a key psychological mechanism explaining some of the improvements obtained after participation in the two sessions. In particular, the increase of the ability to manage emotions by participants may play a role in reducing psychological distress, decreasing the perception of emotional vulnerability, and improving their quality of life. These data could be partially explained by the nature of the 90-minute booster sessions implemented, where adolescents are encouraged to further applying the emotional regulation strategies they have learned, thus consolidating their use in everyday contexts. In this way, the data support the relevance of difficulties in emotional regulation and their importance as a therapeutic target. This has been evidenced in previous studies, where adolescents who initially show no dysfunction may develop psychopathological symptoms due to inflexibility in their emotional regulation (Cobos-Sanchez et al., 2022).

Another possible explanation could be the way in which emotional regulation was assessed. Emotional regulation is understood to be the processes, both internal and external, that are responsible for monitoring, evaluating, and modifying our emotional reactions in order to achieve our goals (Gross, 1998; Koole, 2009; Thompson, 1994) and it has been associated with with anxiety and depression (Aldao et al., 2010). In the present study, the Difficulties in Emotion Regulation Scale was administered, a questionnaire that assesses dimensions such as non-acceptance of emotional responses, difficulty engaging in goal-directed behaviour when distressed, problems controlling impulsive behaviours under emotional pressure, limited access to effective emotion regulation strategies, lack of emotional awareness, and poor emotional clarity. It should be noted that the PROCARE + intervention (Vivas-Fernandez et al., 2023a, b) directly addresses these components of emotional regulation. Through cognitive behavioural therapy (CBT) and mindfulness techniques, adolescents are taught to identify and experience their intense emotions with less distress and to respond in a more adaptive way. This reduces the use of avoidant strategies and promotes effective behaviours in the face of emotional distress.

In summary, these findings suggest that improvements derived from transdiagnostic preventive interventions, as opposed to purely psychoeducational interventions, have been shown to be mediated by an increase in resilience (Jimenez-Vazquez et al., 2024a; Vivas-Fernandez et al., 2024b). However, in the case of improvements achieved following booster sessions within a transdiagnostic intervention framework, changes in emotional regulation appear to play a pivotal role in the obtained benefits. This emphasizes the importance of sustained interventions that promote not only learning, but also the consolidation and maintenance of key regulatory skills in adolescents.

As a line of future research, it would be interesting to investigate the specific emotional regulation mechanisms underlying the improvements observed after the booster sessions. In particular, it would be interesting to examine the extent to which certain emotional regulation factors could act as mediating variables, contributing differentially to the observed effects. This distinction was not addressed in the present study in order to avoid overly complex or overloaded models of mediating variables, which could compromise the interpretability of preliminary analyses. Additionally, this study has limitations, such as a limited sample size. The use of follow-up assessments (including a 13-month assessment) had an impact on sample retainment. Therefore, future studies examining the effects of booster sessions should consider mediation effects using a larger sample size. Another limitation is that the study did not control that changes in this construct precede changes in the outcome variables. Nevertheless, we consider that our analytical approach is consistent with the theoretical framework described regarding the psychological mechanisms involved. While causal inference cannot be fully established without an ideal experimental design, a no-booster session condition was included as a comparison group. Future applied research in clinical and preventive contexts is needed to further explore these aspects. Although the comparisons were independent, the analysis of multiple models might have increased the risk of Type I error.

In conclusion, the possible mediating effects of emotional regulation in reducing levels of risk of emotional problems and symptoms of anxiety and depression, and improvement of quality of life, open up new avenues for understanding the mechanisms of prevention interventions and the maintenance of long-term outcomes.

## Supplementary Information

Below is the link to the electronic supplementary material.

ESM 1 (DOCX 138 KB)

## Data Availability

Data sets generated and/or analyzed during this study are not publicly available due to organizational limitations, but are available from the corresponding author upon reasonable request.
