# Supplementary material for: How Does Booster Work? A Mediation Analysis of the Effects of Booster Sessions in a Transdiagnostic, Selective, Personalised, Preventive Intervention for At-Risk Youth
Source: Res Child Adolesc Psychopathol. 2025 Oct 30;53(12):2053–68. doi: 10.1007/s10802-025-01381-3 (PMC12718278; doi:10.1007/s10802-025-01381-3)
Supplement: Supplementary file 1 — (DOCX 138 KB) [file 10802_2025_1381_MOESM1_ESM.docx]

**How Do Booster Sessions Work? A Mediation Analysis of the Effects of Booster Sessions in a Selective Transdiagnostic Intervention for At-Risk Youth**

**Supplementary Information**

Table S1

Temporal Comparison of Mean Scores Across Study Phases

|  |  | T1: Baseline/ pre‑treatment  Mean (SD) | T2: Post‑treatment  Mean (SD) | T3: 6‑months Follow‑Up  Mean (SD) | T4: 7‑months Follow‑Up  Mean (SD) | T5: 12‑months Follow‑Up  Mean (SD) | T6: 13‑months Follow‑Up  Mean (SD) |
| --- | --- | --- | --- | --- | --- | --- | --- |
|  |  |  |  |  |  |  |  |
| SDQA | G0 | 2.24 (1.70) | 1.48 (1.36) | - | 1.67 (1.24) | - | 2.33 (1.68) |
|  | G1 | 2.67 (1.67) | 1.48 (1.36) | 2.13 (1.42) | 1.00 (0.93) | - | 0.98 (1.14) |
|  | G2 | 2.51 (1.54) | 1.54 (1.10) | 2.05 (1.79) | 1.02 (1.22) | 1.05 (1.21) | 0.74 (0.79) |
| SDQP | G0 | 3.95 (2.84) | 2.76 (1.79) | - | 2.81 (2.34) | - | 3.67 (2.33) |
|  | G1 | 3.58 (2.48) | 2.33 (2.20) | 2.78 (2.19) | 1.70 (1.60) | - | 1.83 (1.71) |
|  | G2 | 3.36 (2.07) | 2.33 (1.83) | 2.62 (1.58) | 1.61 (1.35) | 1.69 (1.20) | 1.59 (1.20) |
| TOTAl- RCADS | G0 | 23.52 (7.99) | 16.67 (7.02) | - | 12.67 (7.41) | - | 18.33 (6.24) |
|  | G1 | 24.03 (7.32) | 16.25 (8.14) | 16.17 (7.64) | 12.15 (6.42) | - | 12.20 (7.34) |
|  | G2 | 25.46 (8.05) | 18.08 (7.21) | 17.69 (10.15) | 13.74 (6.83) | 13.18 (7.91) | 11.41 (6.93) |
| DERS | G0 | 75.24 (17.83) | 69.95 (18.04) | - | 62.90 (17.61) | - | 75.81 (21.37) |
|  | G1 | 82.33 (20.15) | 73.05 (12.13) | 71.48 (15.13) | 64.63 (13.69) | - | 64.70 (16.83) |
|  | G2 | 82.38 (21.76) | 74.03 (17.38) | 74.28 (19.71) | 65.85 (18.15) | 66.56 (20.58) | 62.82 (19.35) |
| CD-RISC-10 | G0 | 24.81 (8.68) | 29.52 (4.92) | - | 29.71 (7.34) | - | 26.33 (4.86) |
|  | G1 | 24.93 (6.09) | 28.68 (6.39) | 29.40 (5.83) | 31.45 (4.48) | - | 31.55 (5.45) |
|  | G2 | 24.71 (6.42) | 29.54 (5.58) | 29.26 (5.73) | 29.79 (5.96) | 29.33 (5.98) | 32.18 (5.77) |

*Note.* Emotional subscale Self-Report SDQ, The Strengths and Difficulties Questionnaire (SDQA); Emotional subscale Parent SDQ: The Strengths and Difficulties Questionnaire (SDQP); CD-RISC: 10-Item Connor-Davidson Resilience; RCADS (Total): Revised Children’s Anxiety and Depression Scale Total score; KIDSCREEN: KIDSCREEN-10 Index; DERS Difficulties in Emotion Regulation Scale, G0: without booster session, G1: One booster session, G2: Two booster session.

**Figure S1**

Significant mediation models *(Without Booster Session vs. One Booster Sessions)*

*a_2_* = 0.29 (0.25)

Change in

CD- RISC (Resilience)

Indirect effect (a_1_ x b_1_)

*ᵝ* = -0.27, *SE* = 0.19, 95% CI [-0.07, 0.01]

Indirect effect (a_2_ x b_2_)

*ᵝ* = -0.02, *SE* = 0.06, 95% CI [-0.08, 0.16]

*c* = -1.15 (0.26) ***

*c*’= -0.90 (0.26) **

*a_1_* = -0.67 (0.25) **

*b_1_* = 0.40 (0.12) **

Change in DERS

(Emotion Regulation)

EG: Experimental Group (without booster session vs 1 booster session)

Change in Emotional Subscale SDQ-A (Self-reported Emotional Risk)

Model 1

*b_2_* = -0.69 (0.13)

*a_2_* = 0.29 (0.25)

Change in

CD- RISC (Resilience)

Indirect effect (a_1_ x b_1_)

*ᵝ* = -0.03, *SE* = 0.10, 95% CI [-0.23, 0.17]

Indirect effect (a_2_ x b_2_)

*ᵝ* = -0.01, *SE* = 0.04, 95% CI [-0.08, 0.11]

*c* = -1.04 (0.28) ***

*c*’= -1.01 (0.30) **

*a_1_* = -0.67 (0.25) **

*b_1_* = 0.50 (0.14)

Change in DERS

(Emotion Regulation)

EG: Experimental Group (without booster session vs 1 booster session)

Change in Emotional Subscale SDQ-P (Emotional Risk reported by parents)

Model 2

*b_2_* = -0.03 (0.14)

*a_2_* = 0.29 (0.25)

Change in

CD- RISC (Resilience)

**Indirect effect (a_1_ x b_1_)**

***ᵝ* = 0.27 *SE* = 0.16, 95% CI [0.05, 0.67]**

Indirect effect (a_2_ x b_2_)

*ᵝ* = 0.06, *SE* = 0.12, 95% CI [-0.07, 0.38]

*c* = 0.62 (0.27) *

*c*’= 0.28 (0.27)

*a_1_* = -0.67 (0.25) **

*b_1_* = -0.461(0.13) **

Change in DERS

(Emotion Regulation)

EG: Experimental Group (without booster session vs 2 booster session)

Change in Total RCADS

(Anxiety and mood symptomatology)

Model 4

*b_2_* = -0.19 (0.12)

*a_2_* = 0.29 (0.25)

Change in

CD- RISC (Resilience)

**Indirect effect (a_1_ x b_1_)**

***ᵝ* = -0.37, *SE* = 0.16, 95% CI [-0.73, -0.09]**

Indirect effect (a_2_ x b_2_)

*ᵝ* = -0.03, *SE* = 0.05, 95% CI [-0.16, 0.05]

*c* = -0.87 (0.24) ***

*c*’= -0.46 (0.20) **

*a_1_* = -0.67 (0.25) **

*b_1_* = 0.55 (0.10) ***

Change in DERS

(Emotion Regulation)

EG: Experimental Group (without booster session vs 2 booster session)

Change in Total RCADS

(Anxiety and mood symptomatology)

Model 3

*b_2_* = 0.12 (0.10)

**Figure S2**

Significant mediation models *(Without Booster Session vs. two Booster Sessions)*

*a_2_* = 1.02 (0.25) ***

Change in

CD- RISC (Resilience)

**Indirect effect (a_1_ x b_1_)**

***ᵝ* = -0.36, *SE* = 0.19, 95% CI [-0.80, -0.08**]

Indirect effect (a_2_ x b_2_)

*ᵝ* = -0.12, *SE* = 0.12, 95% CI [-0.38, 0.07]

*c* = -1.30 (0.23) ***

*c*’= -0.83 (0.22) ***

*a_1_* = -0.77 (2.83) **

*b_1_* = 0.47 (0.10) ***

Change in DERS

(Emotion Regulation)

EG: Experimental Group (without booster session vs 2 booster session)

Change in Emotional Subscale SDQ-A (Self-reported Emotional Risk)

Model 5

*b_2_* = -0.12 (0.11)

*a_2_* = 1.02 (0.25) ***

Change in

CD- RISC (Resilience)

Indirect effect (a_1_ x b_1_)

*ᵝ* = -0.03, *SE* = 0.10, 95% CI [-0.23, 0.17]

Indirect effect (a_2_ x b_2_)

*ᵝ* = -0.03, *SE* = 0.11, 95% CI [-0.26, 0.21]

*c* = -1.14 (0.21) ***

*c*’= -1.08 (0.25) ***

*a_1_* = -0.77 (2.83) **

*b_1_* = 0.40 (0.11)

Change in DERS

(Emotion Regulation)

EG: Experimental Group (without booster session vs 2 booster session)

Change in Emotional Subscale SDQ-P (Emotional Risk reported by parents)

Model 6

*b_2_* = -0.03 (0.12)

*a_2_* = 1.02 (0.25) ***

Change in

CD- RISC (Resilience)

**Indirect effect (a_1_ x b_1_)**

***ᵝ* = 0.35 *SE* = 0.18, 95% CI [0.07, 0.79]**

Indirect effect (a_2_ x b_2_)

*ᵝ* = -0.20, *SE* = 0.12, 95% CI [-0.24, 0.18]

*c* = 1.03 (0.25) ***

*c*’= 0.88 (0.26) **

*a_1_* = -0.77 (2.83) **

*b_1_* = -0.46 (0.11) ***

Change in DERS

(Emotion Regulation)

EG: Experimental Group (without booster session vs 2 booster session)

Change in Total RCADS

(Anxiety and mood symptomatology)

Model 8

*b_2_* = -0.19 (0.12)

*a_2_* = 1.02 (0.25) ***

Change in

CD- RISC (Resilience)

**Indirect effect (a_1_ x b_1_)**

***ᵝ* = -0.42, *SE* = 0.18, 95% CI [-0.81, -0.11]**

Indirect effect (a_2_ x b_2_)

*ᵝ* = -0.01, *SE* = 0.10, 95% CI [-0.24, 0.18]

*c* = -1.03 (0.24) ***

*c*’= -0.61 (0.22) **

*a_1_* = -0.77 (2.83) **

*b_1_* = 0.54 (0.10) ***

Change in DERS

(Emotion Regulation)

EG: Experimental Group (without booster session vs 2 booster session)

Change in Total RCADS

(Anxiety and mood symptomatology)

Model 7

*b_2_* = 0.01 (0.10)
